# Prepared or at-risk? Evaluating live viral vaccine coverage and vaccine immunogenicity before pediatric solid organ transplantation

**DOI:** 10.3389/ti.2026.15958

**Published:** 2026-07-29

**Authors:** Benhur Sirvan Cetin, Caitlin Brammer, William Otto, Kerrigan Perkins, Teresa Ambrosino, Hilary Miller-Handley, Mark Murphy, Grant Paulsen, Emily Cain, Kathleen Campbell, Lara Danziger-Isakov

**Affiliations:** 1 Department of Pediatric Infectious Diseases, Faculty of Medicine, Erciyes University, Kayseri, Türkiye; 2 Division of Infectious Diseases, Cincinnati Children’s Hospital Medical Center, Cincinnati, OH, United States; 3 Department of Pediatrics, University of Cincinnati College of Medicine, Cincinnati, OH, United States; 4 Department of Transplant Administration, Cincinnati Children's Hospital Medical Center, Cincinnati, OH, United States; 5 Division of Infectious Diseases, University of Cincinnati Department of Internal Medicine, Cincinnati, OH, United States; 6 Department of Liver and Intestinal Transplant, Cincinnati Children's Hospital Medical Center, Cincinnati, OH, United States; 7 Division of Gastroenterology, Hepatology and Nutrition, Cincinnati Children's Hospital Medical Center, Cincinnati, OH, United States

**Keywords:** Measles, pediatric solid organ transplantation, serology, vaccination before transplantation, varicella

## Abstract

Pediatric solid organ transplant recipients are at high risk for complications from vaccine-preventable diseases, yet pre-transplant vaccination coverage is often inconsistent. We conducted a retrospective cohort study of 421 pediatric transplant recipients between 2018 and 2024 to evaluate vaccination status and serologic immunity for varicella and measles at the time of initial evaluation, listing, and transplantation. At the time of transplant, 19.2% of patients for varicella and 21.6% for measles were under-vaccinated, with infants having the highest rates of incomplete vaccination. A portion of patients (7.1% for varicella, 8.6% for measles) transitioned from full to incomplete vaccination status during the pre-transplant period, a change associated with a shorter time from evaluation to listing. Kidney recipients had the highest completed vaccination rates (>94%), whereas rates for liver and heart recipients were substantially lower (58%–72%). Among fully vaccinated patients, seronegativity was observed for varicella (23.6%) and measles (13.0%). In conclusion, many pediatric transplant candidates, particularly infants and those awaiting liver or heart transplant, are incompletely vaccinated. A dynamic change in vaccination status from evaluation to transplant highlights an urgent need for continuous monitoring and improved strategies to protect these vulnerable children.

## Graphical Abstract

**Figure:**
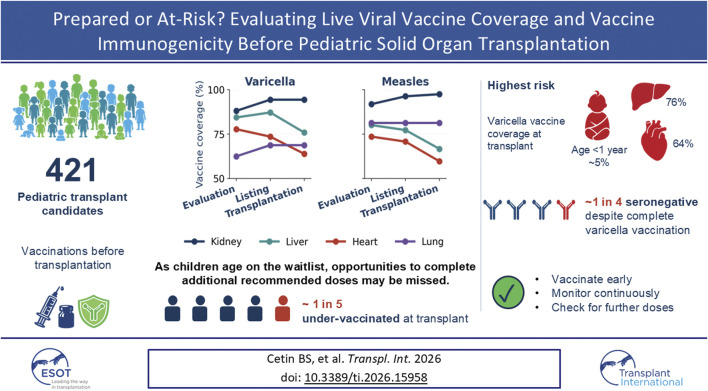

## Introduction

Pediatric solid organ transplant (SOT) recipients are at increased risk for severe complications from vaccine-preventable diseases such as measles, mumps, rubella, and varicella [1]. Because live viral vaccines are often avoided after transplantation, pre-transplant vaccination is essential to protect patients in the vulnerable post-transplant period [2].

Despite recommendations, studies have shown that many SOT candidates remain incompletely vaccinated or are seronegative at the time of transplantation [1, 3]. Contributing factors include early transplantation before completion of routine immunizations, missed opportunities for catch-up vaccination, and inconsistent implementation of pre-transplant vaccination protocols across centers [4]. This gap in protection is especially concerning in light of recent outbreaks of measles and varicella, which can be attributed in part to declining vaccination rates during the COVID-19 pandemic [5, 6]. Transplant recipients are particularly vulnerable during such outbreaks, underscoring the need for optimized pre-transplant immunization strategies [7, 8].

While prior reports have provided valuable cross-sectional assessments, the specific vulnerabilities and dynamic changes in vaccination status during the pre-transplant period remain poorly defined. In this study, we retrospectively evaluated pre-transplant vaccination status and serologic immunity against measles and varicella in a large pediatric SOT cohort to identify gaps in protection and provide insight into modifiable, system-level practices that could sustain high vaccine coverage. Unlike prior cross-sectional reports, this study offers a longitudinal, organ-specific assessment of vaccination coverage at multiple pre-transplant milestones (evaluation, listing, and transplantation) and integrates serologic data to identify immunity gaps.

## Materials and methods

### Study overview and patient selection

A retrospective cohort study was conducted at Cincinnati Children’s Hospital Medical Center (CCHMC), a tertiary care center with active pediatric SOT programs. The study included recipients of a first liver, kidney, heart, lung, intestinal, or multi-visceral transplant between January 1, 2018, and December 31, 2024. Demographic and clinical data were extracted from the electronic medical records, including age at first transplant evaluation, listing and transplantation dates, sex, underlying diagnosis, type of transplanted organ, vaccination records, and serologic results. Vaccination records were obtained from patients’ electronic medical records, which are routinely updated with internal and external vaccine records by the infectious diseases clinic during the standard pre-transplant assessment. Patients were excluded if they lacked vaccination records or were not transplanted at CCHMC.

### Vaccination status

Vaccination status was determined for measles and varicella based on documented vaccination records. *Completed vaccination* was defined separately for measles and varicella as meeting the following criteria for different age groups:

#### Measles

- 6 – <12 months of age: One dose of mumps, measles, and rubella (MMR) vaccine (accelerated regimen)
- ≥12 months - <4 years of age: At least one dose of MMR
- ≥4 years of age: Two or more doses of MMR

#### Varicella

- 9 – <12 months of age: One dose of varicella-zoster virus (VZV) vaccine (accelerated regimen)
- ≥12 months - <4 years of age: At least one dose of vaccine
- ≥4 years of age: Two or more doses of vaccine

The vaccination status of each patient was assessed at three separate time points: initial evaluation, listing for transplant, and date of transplant. Patients could transition from one age group to another between these time points affecting their immunization status. This temporal approach aimed to determine patients' complete vaccination needs as they aged in the pre-transplant period. The lower age limits for starting MMR and VZV vaccinations in patients (≥6 months for MMR and ≥9 months for VZV) were defined according to the guidelines of the American Society of Transplantation Infectious Diseases Community of Practice (AST IDCOP) [2]. Patients younger than the lower age limits at one of the assessment points were accepted as completely vaccinated for that timepoint. While vaccination status was documented, the specific reasons for incomplete vaccination, such as patient or parental vaccine refusal, were not routinely collected in the medical record and thus could not be analyzed. For the one to <4 years age group, completed vaccination was defined as at least one dose of MMR or varicella vaccine to align with age-based eligibility at the time of assessment. While clinical practice at our center aims to administer a second dose if the pre-transplant window allows for the recommended interval between doses, the one-dose threshold was maintained for this analysis to provide a standardized metric of basic coverage. In a sensitivity analysis, complete vaccination for the 1–<4 years group was redefined as two doses and the multivariable models repeated. Pre-transplant seropositivity in this group was also compared between one- and two-dose recipients at the time of testing.

### Vaccine immunogenicity

Pre-transplant vaccine immunogenicity was assessed using commercial chemiluminescent immunoassays (LIAISON, DiaSorin) in routine clinical practice: varicella-zoster virus IgG qualitatively at our institutional laboratory (seropositive >1.0 S/CO) and measles (rubeola) IgG at a reference laboratory (ARUP Laboratories, Salt Lake City, UT; positive ≥16.5 AU/mL, with equivocal results [13.5–16.5 AU/mL] classified as seronegative). Serologic status was analyzed dichotomously (seropositive vs. seronegative); numerical titers were not included. If testing was not performed, immunity status was recorded as unknown.

### Data analysis

Descriptive statistics were used to summarize the data. Categorical variables were expressed as counts and percentages, while continuous variables were summarized using medians and interquartile ranges (IQRs). Comparisons between groups were performed using Chi-square or Fisher’s exact test for categorical variables and Mann–Whitney U or Student’s t-test for continuous variables. A two-sided p-value <0.05 was considered statistically significant. Analyses were conducted using IBM SPSS Statistics (Version 30).

### Ethical approval

This study was approved by the CCHMC Institutional Review Board (IRB ID:2023-0334). Due to the study’s retrospective nature and the use of de-identified data, the requirement for informed consent was waived in accordance with institutional policy.

## Results

### Study cohort

**FIGURE 1:**
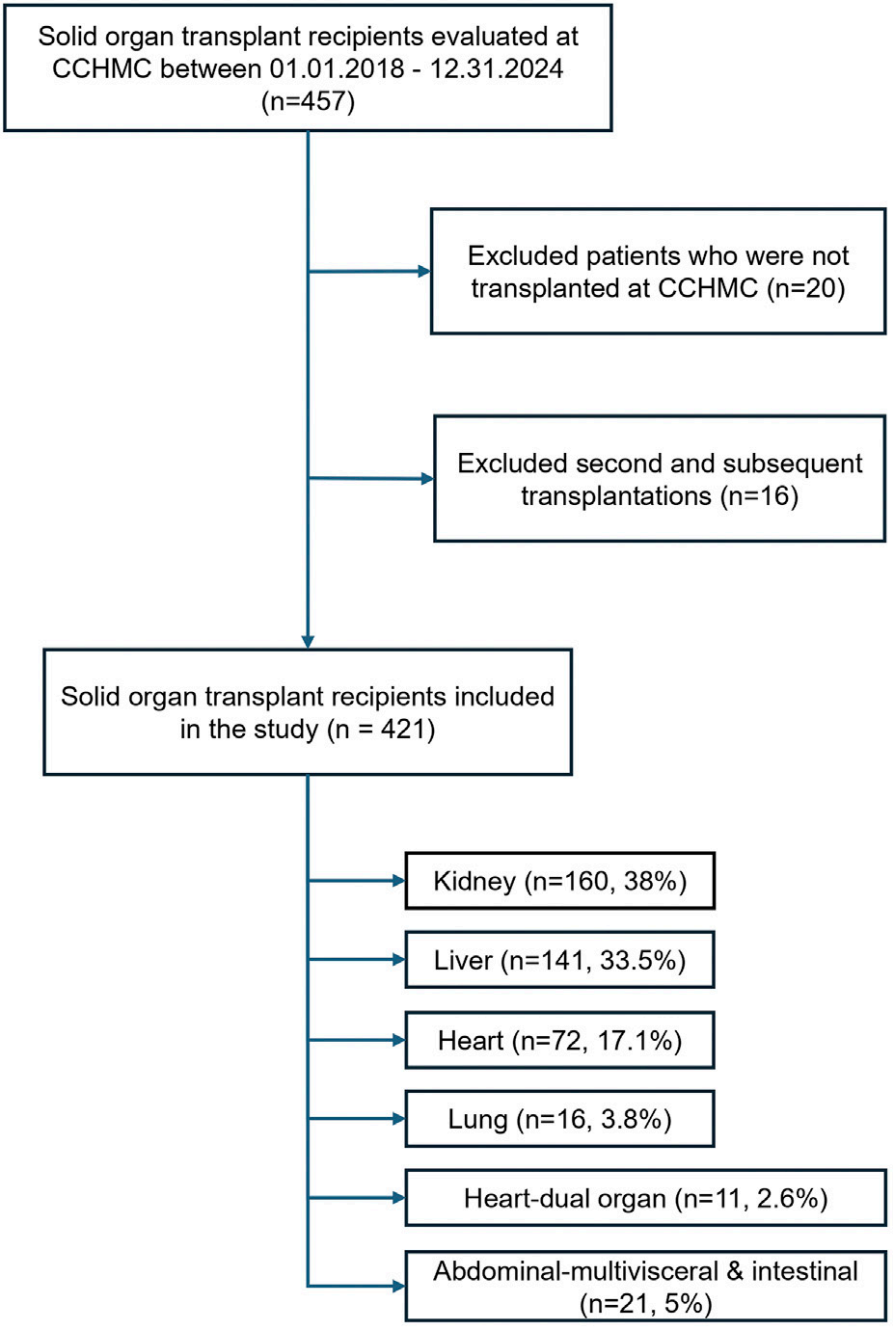
Study cohort and subgroups. The flow diagram illustrates the application of exclusion criteria to define the final study population and shows the distribution by transplanted organ.

During the 7-year study period, a total of 457 patients were evaluated for solid organ transplantation at CCHMC. Twenty patients who were not transplanted at CCHMC and 16 patients who had second or subsequent transplantations were excluded from the study, leaving a total of 421 SOT recipients in the analysis (Figure 1). The distribution of the transplants in the study cohort was 38.0% kidney (160/421), 33.5% liver (141/421), 17.1% heart (72/421), 3.8% lung (16/421), 2.6% heart-dual organ (11/421), and 5% abdominal multi-visceral and intestinal transplant (21/421). The number of transplants per organ type for each calendar year is shown in Supplementary Figure S1.

### Patient demographics

Among 421 patients, 53.7% (226/421) were male. The median age at transplantation was 8 years (range 0.6–38.4 years). Most patients (60.3%, 254/421) were >4 years old, while infants (<12 months) and toddlers (1–<4 years) comprised 13.3% (56/421) and 26.4% (111/421), respectively. Median times between evaluation and listing for transplantation, and between listing and operation were 42 and 114 days, respectively (Table 1).

**TABLE 1:** Demographics of the pediatric solid organ transplant recipients.

Characteristics	All recipients (n = 421)
Age groups at transplant *(n, %)* 0 – <12 months 1 – <4 years ≥4 years	56 (13.3%) 111 (26.4%) 254 (60.3%)
Transplanted organ *(n, %)* Kidney Living donor (kidney) Liver Living donor (liver) Heart Lung Heart-dual organ Abdominal – multivisceral and intestinal	160 (38.0) 60 (37.5)* 141 (33.5) 11 (7.8)* 72 (17.1) 16 (3.8) 11 (2.6) 21 (5.0)
Age at transplant, *median (IQR), years* Kidney Liver Heart Lung Heart-dual organ Abdominal – multivisceral and intestinal	8 (2.2–15.7) 11.4 (3.8–16.2) 2.9 (0.9–11.3) 6.1 (1.3–15.1) 15.2 (10.1–17.4) 20.7 (15.2–22.2) 3.5 (2.2–9.8)
Sex (n,%) Male Female	226 (53.7) 195 (46.3)
Duration between time points, *median (IQR), days* Starting of evaluation to listing for transplantation Listing for transplantation to the operation	42 (20–109.5) 114 (37.5–267.5)

IQR: interquartile range.

* Living-donor frequencies are shown for kidney and liver recipients with percentage within each organ group; the remainder received deceased-donor transplants.

### Vaccination status of the patients

Vaccination status was assessed for the same cohort of 421 recipients at all three time points (evaluation, listing, and transplantation), reflecting longitudinal follow-up of one population rather than separate groups. As age-based eligibility was reassessed at each milestone, the number of completely vaccinated patients could increase through catch-up vaccination or decrease as patients aged into categories requiring additional doses.

#### Varicella

In the cohort, at the time of evaluation, listing, and transplantation, 83.1% (350/421), 86.5% (364/421), and 80.8% (340/421) were fully vaccinated against varicella, respectively (Table 2). Vaccination coverage varied significantly by age. The lowest coverage rates were observed in the 9 – <12 months age group, where none of the 13 patients were vaccinated at evaluation (0/13). Among patients who were 9–<12 months of age at the time of listing and transplantation, only 7.69% (1/13) and 5% (1/20) had received complete vaccination, respectively. Among children aged 1–<4 years, the complete vaccination rate declined from 82.7% (81/98) at initial evaluation to 74.6% (82/110) at the time of transplantation. In contrast, patients aged 4 years or older demonstrated high and improving coverage, with 82.8% (197/238) vaccinated at evaluation and 86.6% (220/254) at transplantation. Despite these rates, 19.2% of the study population (81/421) ultimately underwent transplantation without the recommended age-specified varicella vaccination. Organ-specific analyses revealed that kidney transplant recipients had the highest proportion of completely vaccinated patients regarding varicella (94.4%, 151/160), while the lowest rates were observed among lung (68.8%, 11/16) and heart (63.9%, 46/72) recipients.

**TABLE 2:** Varicella-zoster virus and measles vaccination status of the recipients at different time points.

Complete vaccination rates for varicella						
Study cohort	Evaluation		Listing		Transplantation	
(n = 421)	350/421	(83.1%)	364/421	(86.5%)	340/421	(80.8%)
Age group 0 – <9 months 9 – <12 months 1 – <4 years ≥4 years	72/72 0/13 81/98 197/238	(100%)* (0%) (82.7%) (82.8%)	65/65 1/13 87/100 211/243	(100%)* (7.7%) (87%) (86.8%)	37/37 1/20 82/110 220/254	(100%)* (5%) (74.6%) (86.6%)
Transplanted organ Kidney Liver Heart Lung Heart-dual organ Abdominal-MV. and intestinal	141/160 119/141 56/72 10/16 9/11 15/21	(88.1%) (84.4%) (77.8%) (62.5%) (81.8%) (71.4%)	151/160 123/141 53/72 11/16 9/11 17/21	(94.4%) (87.2%) (73.6%) (68.8%) (81.8%) (81.0%)	151/160 107/141 46/72 11/16 9/11 16/21	(94.4%) (75.9%) (63.9%) (68.8%) (81.8%) (76.2%)

Complete vaccination rates for measles						
Study cohort	Evaluation		Listing		Transplantation	
(n = 421)	350/421	(83.1%)	352/421	(83.6%)	330/421	(78.4%)
Age group 0 – <6 months 6 – <12 months 1 – <4 years ≥4 years	54/54 1/30 81/98 214/238	(100%)* (3.3%) (82.7%) (89.9%)	42/42 4/36 88/100 218/243	(100%)* (11.1%) (88%) (89.7%)	15/15 3/42 83/110 229/254	(100%)* (7.1%) (75.5%) (87.4%)
Transplanted organ Kidney Liver Heart Lung Heart-dual organ Abdominal-MV. and intestinal	147/160 113/141 53/72 13/16 9/11 15/21	(91.9%) (80.1%) (73.6%) (81.3%) (81.8%) (71.4%)	154/160 109/141 51/72 13/16 9/11 16/21	(96.3%) (77.3%) (70.8%) (81.3%) (81.8%) (76.2%)	156/160 94/141 43/72 13/16 9/11 15/21	(97.5%) (66.7%) (59.7%) (81.3%) (81.8%) (71.4%)

The same 421 recipients were assessed at all time points; counts may rise with catch-up vaccination or fall as patients age into categories requiring additional doses.

* For each assessment at a given time point, patients who were not eligible for the vaccine because of age were defined as having a complete vaccination status.

MV: multivisceral.

#### Measles

Complete measles vaccination rates at the time of evaluation, listing, and transplantation were 83.1%, 83.6%, and 78.3%, respectively (Table 2). The lowest rates of coverage were observed in the 6 – <12 months age group, with only 3.3% (1/30) fully vaccinated at evaluation. In patients who were 6 - <12 months of age at listing and transplantation time, only 11.1% (4/36), and 7.1% (3/42) of them had received complete vaccination, respectively. Among children aged 1 – <4 years, the rate of complete vaccination declined from 82.7% (81/98) at initial evaluation to 75.45% (83/110) at the time of transplantation. In contrast, patients older than 4 years consistently had high coverage rates, with 89.9% (214/238) and 87.4% (229/254) at evaluation and transplantation, respectively. Notably, 21.6% of the entire study population (91/421) underwent transplantation without receiving the complete recommended measles vaccination. The highest pre-transplant measles vaccination rate was found among kidney recipients (97.8%, 156/160), whereas patients undergoing liver (66.7%, 94/141) and heart (59.7%, 43/72) transplants exhibited the lowest vaccination rates.

### Changes in vaccination status from the first evaluation to transplantation

**FIGURE 2:**
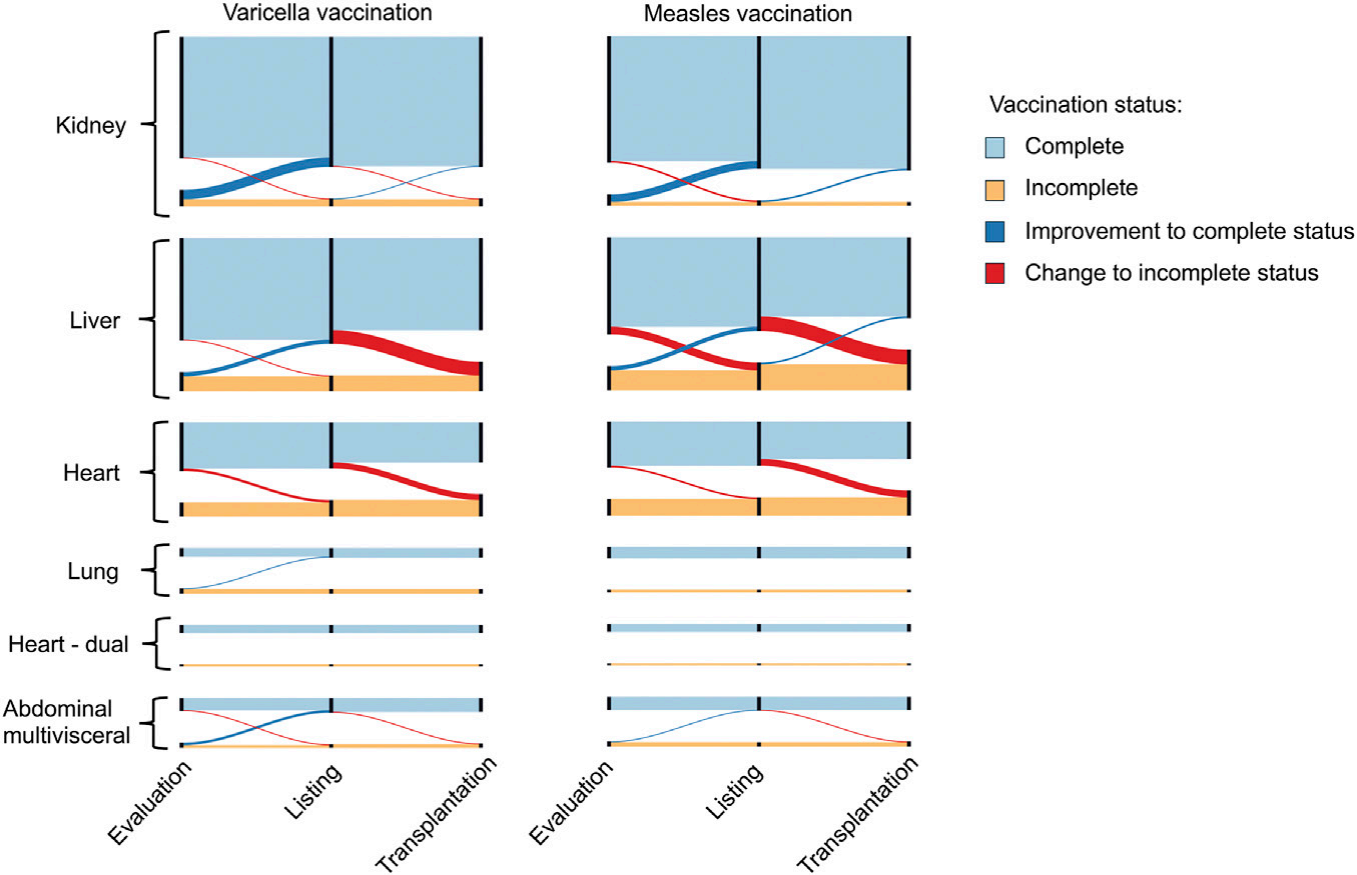
Sankey diagram illustrating changes in vaccination status for varicella and measles from evaluation to transplantation, stratified by organ group. Blue bands represent patients with complete vaccination status, while orange/red bands indicate incomplete status. The width of each flow is directly proportional to the number of patients at each time point, including evaluation, listing, and transplantation. Darker shades highlight transitions, where individuals change from complete to incomplete (dark red) or from incomplete to complete (dark blue). Substantial transitions in complete vaccination were particularly evident among liver and heart transplant recipients, for both varicella and measles.

During follow-up, changes in pre-transplant vaccination status were observed in a subset of patients (Figure 2). For varicella, 7.1% (30/421) of patients transitioned from full to incomplete status, most frequently among heart transplant recipients (10/72, 13.9%) and liver transplant recipients (17/141, 12.1%), primarily based on changes in age category. Conversely, 4.8% (20/421) improved from incomplete to complete, primarily among kidney (11/160, 6.9%), liver (5/141, 3.5%), and lung (1/16, 6.3%) candidates, with no improvement observed in heart and heart-dual organ recipients. Similarly, for measles, 8.6% (36/421) transitioned to incomplete vaccination status, with the highest rates among liver (25/141, 17.7%) and heart (10/72, 13.9%) recipients. Improvement occurred in 3.8% (16/421), most commonly among kidney (9/160, 5.6%) and liver (6/141, 4.3%) transplant candidates, while no improvement was noted in lung, heart, or heart-dual organ recipients.

### Factors for incomplete vaccination

To identify patient characteristics associated with the risk of incomplete vaccination status at the time of transplantation, we compared demographics and clinical factors (Table 3). In this analysis, only cases exceeding the lower age limit of the relevant vaccine were included as vaccine-eligible (384 patients for varicella, and 406 patients for measles vaccination).

**TABLE 3:** Comparison of characteristics among vaccine-eligible transplant recipients by vaccination status at the time of transplantation.

Variables	Varicella (n = 384)*					Measles (n = 406)**				
​	Completely vaccinated (n = 303)		Incompletely vaccinated (n = 81)		P Value	Completely vaccinated (n = 315)		Incompletely vaccinated (n = 91)		P Value
Sex [*n,(%)*] Male Female	171 132	(56.4) (43.6)	42 39	(51.9) (48.1)	0.529	179 136	(56.8) (43.2)	41 50	(45.1) (54.9)	0.056
Organ group [*n, (%)*] Kidney Liver Heart Lung Heart-dual Abdominal-MV. and intestinal	151 81 36 11 9 15	(49.8) (26.7) (11.9) (3.6) (3.0) (5.0)	9 34 26 5 2 5	(11.1) (42.0) (32.1) (6.2) (2.5) (6.2)	**<0.05** **<0.05** **<0.05**	156 84 38 13 9 15	(49.5) (26.7) (12.1) (4.1) (2.9) (4.8)	4 47 29 3 2 6	(4.4) (51.6) (31.9) (3.3) (2.2) (6.6)	**<0.05** **<0.05** **<0.05**
Age [median years, (IQR)] Evaluation Listing Transplant	10.3 10.5 10.9	(2.8–15.3) (3.1–15.6) (3.6–16.2)	1.1 1.2 1.7	(0.5–13.5) (0.6–14.1) (1.0–14.9)	**<0.001** **<0.001** **<0.001**	10.4 10.7 11.1	(2.8–15.4) (3.1–15.8) (3.6–16.3)	0.7 0.8 1.2	(0.4–3.6) (0.5–4.0) (0.8–4.3)	**<0.001** **<0.001** **<0.001**
Age group at evaluation [*n, (%)*] <1 year 1 – <4 years ≥4 years	9 87 207	(3.0) (28.7) (68.3)	39 11 31	(48.1) (13.6) (38.3)	**<0.05** **<0.05** **<0.05**	11 88 216	(3.5) (27.9) (68.6)	59 10 22	(64.8) (11.0) (24.2)	**<0.05** **<0.05** **<0.05**
Duration [median days, (IQR)] Evaluation to listing Listing to transplant	58 138	(26.0–144.0) (39.0–297.0)	28 107	(15.0–78.0) (51.5–213.0)	**<0.001** 0.370	59 136	(26.0–140.0) (40.0–298.0)	23 97	(12.0–43.0) (41.0–169.0)	**<0.001** 0.057

* Patients who were transplanted before 9 months of age are excluded from varicella vaccination-related analysis in this table.

** Patients who were transplanted before 6 months of age are excluded from the measles vaccination-related analysis in this table.

IQR, interquartile range; MV, multivisceral. Bold values indicate statistically significant differences (p < 0.05).

The distribution of organ types differed significantly for both varicella and measles (p < 0.05 for both). For varicella, liver and heart candidates were disproportionately represented in the incompletely vaccinated group, accounting for 42.0% (34/81) and 32.1% (26/81) of this cohort, respectively. In contrast, kidney recipients comprised nearly half of the fully vaccinated group (49.8%, 151/303) but only 11.1% (9/81) of the incompletely vaccinated group.

For measles, the disparity between organ groups was even more pronounced. Liver and heart candidates together constituted 83.5% of the incompletely vaccinated cohort (51.6% [47/91] and 31.9% [29/91], respectively). Conversely, kidney recipients accounted for 49.5% (156/315) of fully vaccinated patients but only 4.4% (4/91) of those who were incompletely vaccinated, highlighting a strong association between organ type and the likelihood of completing the recommended measles vaccination series before transplantation. While the lung transplant group had the second-lowest observed rate of complete vaccination (68.8%) for varicella, due to the small number of lung transplants, the difference between their vaccination rates in vaccine-eligible patients was not statistically significant.

Age was also a significant factor; children under 1 year of age at the first evaluation accounted for nearly half of those incompletely vaccinated for varicella (48.1%) and the majority of those incompletely vaccinated for measles (64.8%) at the time of transplantation. Consequently, the median ages at evaluation, listing, and transplant were all significantly lower in the incompletely vaccinated groups (p < 0.001 for all, Table 3). The duration between initial evaluation and listing was significantly shorter for patients in the incompletely vaccinated groups for both varicella (median 28 vs. 58 days; p < 0.001) and measles (median 23 vs. 59 days; p < 0.001). In multivariable analysis (Supplementary Tables S1, S2), non-kidney candidates and younger patients had significantly higher odds of incomplete vaccination (p < 0.05). Conversely, the evaluation-to-listing interval lost significance after adjustment. This suggests organ type captures the clinical urgency driving shorter timelines, while young age remains a distinct, independent barrier. In a sensitivity analysis applying a stricter two-dose definition for the 1–<4 years group, non-kidney organ type and younger age remained independent predictors for both vaccines (Supplementary Tables S3, S4). No significant differences were observed based on sex, year of transplantation, or the duration from listing to transplantation.

### Vaccine immunogenicity

Serological tests were not performed on all patients at each assessment point or vaccination. Overall, varicella and measles serology were available for 94.3% (397/421) and 57.9% (244/421) of patients, respectively. To minimize the effect of passive maternal antibodies, all tests from patients under 12 months of age were excluded from comparative analysis. In completely vaccinated recipients, pre-transplant seropositivity was 76.4% (217/284) for varicella and 87.0% (167/192) for measles.

Among 1–<4-year-olds with serology, most had received one dose (74.7% varicella, 81.5% measles). Varicella seropositivity was lower after one than two doses (67.7% [44/65] vs. 95.5% [21/22]; p = 0.010), with 9 of 10 initially seronegative children seroconverting after the second dose; measles was already immunogenic after one dose (95.5% vs. 100%; p = 1.0). Among fully vaccinated older children (≥4 years), differences in seropositivity rates for measles were observed across the organ groups (p = 0.007, Table 4). Post-hoc analysis revealed that the seronegativity rate in kidney recipients (25.4%) was significantly higher than the rates in both liver (0.0%) and heart (5.0%) recipients (p < 0.05). Additionally, older kidney recipients were significantly more likely to be seronegative than their younger counterparts (93.1% vs. 74.6%, p < 0.05). For varicella, a trend toward higher seropositivity was observed in liver recipients (88.9%) compared to kidney (72.9%) and heart (66.7%) recipients; however, this difference was not statistically significant (p = 0.057).

**TABLE 4:** Comparison of the most recent pre-transplantation serological immunity and vaccination status at the time of the test.*

​		Varicella (n = 305)						Measles (n = 201)					
Age group at the test time		1 – <4 years (n = 87)		≥4 years (n = 21)		≥4 years (n = 197)		1 – <4 years (n = 54)		≥4 years (n = 9)		≥4 years (n = 138)	
Vaccination status at the test time		Completely vaccinated (≥1 dose)		Incompletely vaccinated (1 dose)		Completely vaccinated (≥2 doses)		Completely vaccinated (≥1 dose)		Incompletely vaccinated (1 dose)		Completely vaccinated (≥2 doses)	
Transplanted organ	​	n	(%)	n	(%)	n	(%)	n	(%)	n	(%)	n	(%)
Kidney	SN SP	15 38	(28.3) (71.7)	4 4	(50) (50)	26 70	(27.1) (72.9)	2 27	(6.9) **(93.1)** ^‡^	0 2	(100)	16 47	**(25.4)** ^†^ **(74.6)** ^‡^
Liver	SN SP	6 17	(26.1) (73.9)	2 5	(28.6) (71.4)	5 40	(11.1) (88.9)	0 14	(100)	0 3	(100)	0 25	**(100)** ^§^
Heart	SN SP	1 5	(16.7) (83.3)	1 1	(50) (50)	10 20	(33.3) (66.7)	0 6	(100)	0 3	(100)	1 19	(5) (95)
Other	SN SP	0 5	(100)	1 3	(25) (75)	4 22	(15.4) (84.6)	0 5	(100)	0 1	(100)	6 24	(20) (80)
Overall	SN SP	22 65	(25.3) (74.7)	8 13	(38.1) (61.9)	45 152	(22.8) (77.2)	2 52	(3.7) (96.3)	0 9	(100)	23 115	(16.7) (83.3)

* This analysis excludes two groups: patients who had never received the relevant vaccine and any serology tests performed before 12 months of age.

SN, seronegative; SP, seropositive. Bold values indicate proportions that differed significantly in the comparisons specified in footnotes †, ‡, and §.

^†^ p = 0.007 for the comparison of kidney vs. all other organ recipients (including liver and heart) for measles seronegativity among completely vaccinated patients aged ≥4 years.

^‡^ p = 0.048 for the comparison of measles seropositivity in completely vaccinated kidney recipients aged 1–<4 years (% 93.1%) vs. ≥4 years (74.6%).

^§^ p = 0.007 for the comparison of liver recipients vs. all other organ recipients for measles seropositivity in that age group.

## Discussion

In this study, we assessed the vaccination status and serologic response against measles and varicella in a large group of pediatric SOT recipients. Ensuring immunization before transplantation is important to minimize the risk of vaccine-preventable infections in immunocompromised children [2, 9]. Our cohort demonstrated high overall pre-transplant vaccination rates compared to previously reported studies. For instance, our completion rates for varicella (80.8%) and measles (78.4%) are high compared to findings from a single-center cohort (54.9% and 69.2%, respectively) and a larger multicenter study (34% and 36%, respectively) [1, 3]. These results may reflect institutional policies that emphasize early consultation with infectious disease specialists and structured vaccine tracking for all transplant candidates.

Despite longstanding recommendations to complete live viral vaccinations prior to transplantation, our study found that a considerable proportion of pediatric candidates remained incompletely vaccinated at the time of transplantation. Our results are consistent with a broad body of evidence documenting similar, and in some cases more pronounced, gaps in care across various pediatric transplant populations. A multicenter European reference network survey revealed a systemic challenge, with complete immunization coverage of at least 80% of patients being achieved in only 57% of participating pediatric SOT programs [10]. A study from the US showed that only about half (54.9%) of pediatric transplant candidates had a complete varicella vaccination history, and only 69.2% had completed their age-appropriate measles vaccinations [1]. A study focused on European pediatric liver transplant programs reported that nearly 35% of the candidates were under-vaccinated for varicella [11]. A key reason identified for these gaps is often the insufficient time available for vaccination before a transplant is required.

### The dynamic changes in pre-transplant vaccination status

A distinctive strength of our study lies in its organ-specific analysis and longitudinal tracking of vaccination status from initial evaluation to the time of transplantation. While other reports have provided valuable snapshots of incomplete vaccination across SOT populations, our longitudinal analysis, to our knowledge, is the first to document the transition of status systematically. This longitudinal approach revealed that overall vaccine coverage declined during the pre-transplant period, with a notable subset of patients—7.1% for varicella and 8.6% for measles—transitioning from complete to incomplete status rather than progressing toward schedule completion (Figure 2). As status was re-evaluated by age at each milestone, completely vaccinated counts could rise with catch-up vaccination or fall as patients aged into stricter requirements; the net decline thus reflects reclassification rather than loss of previously administered vaccines. The transition in vaccination status was most prominent in liver and heart transplant candidates. These shifts were primarily driven by age-based eligibility changes, highlighting the importance of continued, dynamic assessment of immunization status throughout the waitlist period.

### Effects of patient age on incomplete vaccination

Notably, the lowest vaccination rates in our cohort were observed among infants aged 9 to <12 months. These low rates highlight a critical vulnerability in the youngest candidates, a finding that is strongly corroborated by other research. One study found that a staggering 81% of infants aged 6–11 months with documented vaccination status had not received the first dose of the varicella vaccine prior to transplantation [12]. The same study concluded that this specific age group accounted for nearly half (47%) of missed immunization opportunities for the first vaccine dose. Crucially, our multivariable analysis confirmed that younger age is an independent predictor of incomplete vaccination, distinct from the type of organ transplant. Although our criteria for toddlers (1 to <4 years) required only one dose for 'complete' status, the decline in coverage observed as patients age underscores the difficulty of completing the full two-dose series. This definition did not affect our conclusions on sensitivity analysis, but its serologic impact was vaccine-specific: a single varicella dose was seroprotective in only 67.7% (vs. 95.5% after two doses), whereas one measles dose sufficed (95.5%), supporting prioritization of two-dose varicella completion before transplantation. Nonetheless, where sufficient time exists before transplantation, these candidates should still complete the full two-dose measles schedule recommended by AST IDCOP [2]. Guidelines for transplant candidates suggest accelerating vaccination, including early initiation (MMR from 6 months, varicella from 9 months) and early completion (second dose before age 4). Our data, and those of others, demonstrate that uptake of the early first dose is lagging. Low vaccine uptake in this population is further demonstrated by the European network survey, which found that 39% of transplant programs reported never prescribing an accelerated schedule for live-attenuated vaccines to infants [10].

Specific reasons for incomplete vaccination were not systematically recorded; however, the decline among younger candidates likely reflects urgent transplantation, temporary medical contraindications, and missed opportunities for accelerated dosing. Broader implementation of accelerated schedules, both early initiation and completion, improved documentation and standardized tracking could help clarify these barriers and enhance preparedness.

### Organ-specific considerations for incomplete vaccination

In contrast to the kidney group, where we saw rates of full vaccination exceeding 94% for both measles and varicella, rates at transplant were considerably lower for liver (66.7% for measles, 75.9% for varicella) and heart recipients (59.7% for measles, 63.9% for varicella). One of the key findings of our study was the unique vulnerability of heart transplant candidates as the existing literature is limited by publication era or study design [1, 13, 14]. In the multicenter observational study from Europe that included 430 pediatric liver transplant recipients, varicella and measles vaccination coverage rates before transplantation were reported as 65.2% and 81.1% respectively [11]. Again, a multicenter cohort of 281 pediatric SOT recipients (96% liver) from the US reported similarly low pre-transplant vaccination rates of 36% for MMR and 34% for varicella [3].

The underlying reason for increased risk in liver and heart candidates appears to be the urgency of their clinical course. The challenge of a shortened timeline is directly supported by the survey of European transplant centers, where “insufficient time before transplant” was cited by 86% of programs as the primary reason for incomplete immunization [10]. In contrast, the relative stability and higher vaccination rates we observed among kidney transplant recipients may reflect their longer and more predictable pre-transplant course afforded by the availability of dialysis. In our analysis, the evaluation-to-listing interval lost significance after adjustment, suggesting organ type effectively proxies for the clinical urgency and limited vaccination window characterizing liver and heart transplantation. Liver candidates lack a comparable bridging therapy, and while pediatric heart candidates may be stabilized with mechanical circulatory support devices, their clinical course often remains urgent. This fundamental difference in pre-transplant management directly impacts the window of opportunity for completing immunization schedules, positioning kidney recipients at a distinct advantage for vaccination administration.

### Vaccine immunogenicity in the patients

Our cohort’s overall seropositivity rates in completely vaccinated children—76.4% for varicella and 87.0% for measles—appear more favorable but still demonstrate that a significant percentage of children who are up to date on vaccinations lack detectable antibodies. This discordance between vaccination records and humoral immunity is a well-documented challenge in transplant populations with one study of pediatric kidney candidates reporting seropositivity rates were as low as 59.0% for measles and 43.6% for varicella despite being fully vaccinated [15]. Another study found 50% seropositivity among children aged 7 years or older with two documented varicella vaccine doses [1].

This seropositivity gap was influenced by organ type and other clinical factors. In a multicenter study focused on pediatric liver transplant candidates, 53.1% were seropositive for varicella and 62.9% for measles before transplant [11].

In our cohort, older, completely vaccinated kidney recipients showed lower measles seropositivity than other organ groups. Although we did not collect renal-function laboratory values or dialysis data and did not perform any analysis relating these factors to serologic status, prior literature offers a plausible context: chronic kidney disease (CKD) and the uremic state have been associated with immune dysfunction and impaired vaccine responses [16]. Furthermore, factors such as the dialysis modality and a more rapid waning of vaccine-induced antibodies are documented challenges in this population [15, 16]. This observation is not limited to pediatrics, as studies in adult transplant candidates have also found that kidney recipients can be more likely to be seronegative despite vaccination compared to other transplant groups [17]. These mechanisms were not tested here and remain hypotheses for future, appropriately designed studies.

The discrepancy between vaccination history and measurable seropositivity raises critical questions about how best to assess protection before transplant. While national guidelines for the general pediatric population often consider two documented vaccine doses as sufficient evidence of immunity regardless of serology, transplant-specific guidelines recommend a more cautious approach due to the high risks in this population [1, 18]. For instance, the AST IDCOP guidelines recommend considering a third dose of MMR or varicella vaccine for transplant candidates who remain seronegative after completing their initial series [2]. However, the decision to revaccinate based on titers is complex, as serology measures only humoral immunity, and protection may persist through T-cell-mediated responses even with undetectable antibody levels [18, 19]. Ultimately, incorporating serologic screening into pre-transplant evaluations for select patients—especially those in high-risk groups, such as those with CKD—could help mitigate the risk of post-transplant vaccine-preventable diseases. The assessment of cellular immunity—such as Lymphocyte Transformation Testing (LTT)—and benchmarking against antibody persistence for non-live vaccines (e.g., tetanus) in seronegative candidates represent important targets for future research aimed at refining the assessment of pre-transplant vaccination status in children.

### Limitations of the study

This study has some limitations. First, its retrospective design carries an inherent risk of information bias, particularly regarding vaccination histories from outside institutions. However, to mitigate this, our center has a standardized practice of collecting and integrating external vaccine records into the patient’s electronic medical record. Additionally, we could not account for unmeasured confounders, such as socioeconomic factors, parental vaccine hesitancy, or exposure to blood products or intravenous immunoglobulin—which can temporarily defer live viral vaccination. These unmeasured factors may influence vaccine administration. Our dataset did not include renal function laboratory values or dialysis details (duration or modality), and we did not perform any analyses relating these factors to serologic status. The lower seropositivity observed among kidney recipients is therefore a descriptive finding only; the contribution of the uremic state and dialysis-related factors, while biologically plausible and supported by prior literature, could not be evaluated in this study and should be addressed in future work. Another limitation is the incomplete serologic data for measles, available for only 58% of the cohort. Routine institutional screening for measles was implemented only during the final 2 years of the study period. Despite this, the available data still provides valuable insights into seropositivity gaps. Our reliance on serology provides information solely on humoral immunity, although cellular immunity also plays a protective role. Therefore, seronegativity does not definitively equate to a lack of protection. Nevertheless, serologic testing is currently the clinical standard for assessing the risk of vaccine-preventable diseases and guiding clinical decisions, such as the administration of additional vaccine doses. Finally, as a single-center study at a tertiary referral institution, our results—particularly our higher-than-average vaccination rates—may not be fully generalizable to other settings. However, the consistent protocols at a single center allowed for a detailed longitudinal analysis that is a unique strength of this study. Furthermore, the core challenges identified by this study, such as the impact of clinical urgency on vaccination timelines and the presence of organ-specific immunity gaps, are fundamental issues likely applicable to other pediatric transplant centers.

In conclusion, our longitudinal analysis uniquely reveals that the risk for incomplete vaccination is a dynamic process likely reflecting an overlap in system and clinical barriers. Shifts in care from primary to specialty providers, limited time for vaccination in acutely ill children, and variability in infectious disease involvement may contribute. A patient’s vaccination status can change during the pre-transplant period as they age, highlighting the need for continuous monitoring. Early infectious disease consultation and standardized vaccine tracking within transplant workflows could help reduce missed opportunities and improve coverage. We identified infants and those awaiting liver or heart transplantation as the most vulnerable subgroups, where shorter pre-transplant timelines directly correlate with a failure to maintain or complete vaccination schedules. Furthermore, our serologic data confirm that the number of vaccine doses received is not a guarantee for seropositivity. Therefore, these combined challenges underscore the urgent need for proactive, continuous vaccine management, including early implementation of expedited protocols and consideration of serologic screening, to ensure this high-risk population is truly protected.

## Data Availability

The data analyzed in this study is subject to the following licenses/restrictions: the datasets analyzed during this study are not publicly available because of patient privacy concerns and institutional data protection policies. Requests to access these datasets should be directed to Lara.Danziger-Isakov@cchmc.org.
