# Supplementary material for: Prepared or at-risk? Evaluating live viral vaccine coverage and vaccine immunogenicity before pediatric solid organ transplantation
Source: Transpl Int. 2026 Jul 29;39:15958. doi: 10.3389/ti.2026.15958 (PMC13461561; doi:10.3389/ti.2026.15958)
Supplement: Supplementary file 1 [file Table1.docx]

**Table S1.** Univariable and multivariable logistic regression analysis of factors associated with incomplete varicella vaccination status at the time of transplantation.

|  | Varicella vaccination status | | Univariate | | Multivariable | | |
| --- | --- | --- | --- | --- | --- | --- | --- |
|  | Complete | Incomplete | OR (%95Cl) | P value | OR (%95Cl) | P value |  |
| **Sex, n(%)** |  |  |  |  |  |  |  |
| Female | 132 (43.6) | 39 (48.1) | Reference Category |  |  |  |  |
| Male | 171 (56.4) | 42 (51.9) | 0.8 (0.50: 1.35) | 0.461 |  |  |  |
| **Transplanted organ, n(%)** |  |  |  |  |  |  |  |
| Kidney | 151 (49.8) | 9 (11.1) | Reference Category |  |  |  |  |
| Liver | 81 (26.7) | 34 (42.0) | 7.0 (3.22: 15.40) | **<0.001** | 6.7 (2.81: 16.35) | **<0.001** |  |
| Heart | 36 (11.9) | 26 (32.1) | 12.1 (5.22: 28.08) | **<0.001** | 13.8 (5.35: 35.69) | **<0.001** |  |
| Lung | 11 (3.6) | 5 (6.2) | 7.6 (2.17: 26.69) | **0.001** | 10.4 (2.82: 38.48) | **<0.001** |  |
| Heart-dual | 9 (3.0) | 2 (2.5) | 3.7 (0.7: 19.87) | 0.123 | 6.9 (1.21: 39.90) | **0.029** |  |
| Abdominal-multivisceral & intestinal | 15 (5.0) | 5 (6.2) | 5.5 (1.65: 18.85) | **0.005** | 5.2 (1.45: 19.08) | **0.011** |  |
| **Age at transplant**  **(years), median(IQR)** | 10.9 (12.58) | 1.7 (13.82) | 0.9 (0.90: 0.97) | **0.002** | 0.9 (0.90: 0.98) | **0.005** |  |
| **Interval from evaluation to listing (days), median(IQR)** | 58 (118) | 28 (63) | 0.99 (0.993: 0.999) | **0.01** | 1.0 (0.99: 1.00) | 0.555 |  |
| **Interval from listing to transplant (days), median(IQR)** | 138 (258) | 107 (161.5) | 1 (0.99: 1) | 0.382 |  |  |  |

Patients younger than 9 months at the time of vaccination assessment were excluded from Varicella analysis. Kidney transplant recipients served as the reference category for all organ-related comparisons. Odds ratios (ORs) represent the likelihood of incomplete vaccination status compared with the reference category. Continuous variables are presented as median and interquartile range (IQR). Categorical variables are presented as n (%). Statistically significant p-values are shown in bold.
